# Lifestyle and chronic kidney disease: A machine learning modeling study

**DOI:** 10.3389/fnut.2022.918576

**Published:** 2022-07-22

**Authors:** Wenjin Luo, Lilin Gong, Xiangjun Chen, Rufei Gao, Bin Peng, Yue Wang, Ting Luo, Yi Yang, Bing Kang, Chuan Peng, Linqiang Ma, Mei Mei, Zhiping Liu, Qifu Li, Shumin Yang, Zhihong Wang, Jinbo Hu

**Affiliations:** ^1^Department of Endocrinology, The First Affiliated Hospital of Chongqing Medical University, Chongqing, China; ^2^Laboratory of Reproductive Biology, School of Public Health and Management, Chongqing Medical University, Chongqing, China; ^3^School of Public Health and Management, Chongqing Medical University, Chongqing, China; ^4^Department of Clinical Nutrition, The First Affiliated Hospital of Chongqing Medical University, Chongqing, China; ^5^The Chongqing Key Laboratory of Translational Medicine in Major Metabolic Diseases, The First Affiliated Hospital of Chongqing Medical University, Chongqing, China

**Keywords:** lifestyle, chronic kidney disease, machine learning, scoring system, cohort study

## Abstract

**Background:**

Individual lifestyle varies in the real world, and the comparative efficacy of lifestyles to preserve renal function remains indeterminate. We aimed to systematically compare the effects of lifestyles on chronic kidney disease (CKD) incidence, and establish a lifestyle scoring system for CKD risk identification.

**Methods:**

Using the data of the UK Biobank cohort, we included 470,778 participants who were free of CKD at the baseline. We harnessed the light gradient boosting machine algorithm to rank the importance of 37 lifestyle factors (such as dietary patterns, physical activity (PA), sleep, psychological health, smoking, and alcohol) on the risk of CKD. The lifestyle score was calculated by a combination of machine learning and the Cox proportional-hazards model. A CKD event was defined as an estimated glomerular filtration rate <60 ml/min/1.73 m^2^, mortality and hospitalization due to chronic renal failure, and self-reported chronic renal failure, initiated renal replacement therapy.

**Results:**

During a median of the 11-year follow-up, 13,555 participants developed the CKD event. Bread, walking time, moderate activity, and vigorous activity ranked as the top four risk factors of CKD. A healthy lifestyle mainly consisted of whole grain bread, walking, moderate physical activity, oat cereal, and muesli, which have scored 12, 12, 10, 7, and 7, respectively. An unhealthy lifestyle mainly included white bread, tea >4 cups/day, biscuit cereal, low drink temperature, and processed meat, which have scored −12, −9, −7, −4, and −3, respectively. In restricted cubic spline regression analysis, a higher lifestyle score was associated with a lower risk of CKD event (*p* for linear relation < 0.001). Compared to participants with the lifestyle score < 0, participants scoring 0–20, 20–40, 40–60, and >60 exhibited 25, 42, 55, and 70% lower risk of CKD event, respectively. The C-statistic of the age-adjusted lifestyle score for predicting CKD events was 0.710 (0.703–0.718).

**Conclusion:**

A lifestyle scoring system for CKD prevention was established. Based on the system, individuals could flexibly choose healthy lifestyles and avoid unhealthy lifestyles to prevent CKD.

## Introduction

Chronic kidney disease (CKD) is a progressive condition, which affects approximately 10% of adults worldwide (1). As a growing public health problem, CKD not only increases the risk of end-stage kidney disease (ESRD) and cardiovascular disease (1) but also exerts a major effect on global mortality (2). A healthy lifestyle is important for the preservation of kidney function, while the quality of evidence-based lifestyle recommendations for CKD remains weak.

On the basis of cardiovascular risk, a healthy lifestyle was defined as eating a high-quality diet, moderate- to vigorous-intensity physical activity (PA), modest body mass index, never smoking, and drinking alcohol in moderation (3–8). Although CKD and cardiovascular disease are closely connected by similar epidemiological risk factors and mechanisms, a straightforward extrapolation of cardiovascular healthy lifestyle to CKD prevention needs to be cautiously handled (9). Recently, the relationship between CKD incidence and certain lifestyle, such as dietary patterns (10–12), physical activity (13, 14), alcohol consumption (15–17), cigarette smoking (18, 19), sleep (20), and psychological health (21, 22), has been investigated in some studies with a limited sample size, and the results were equivocal. Furthermore, individual lifestyle varies greatly in the real world. Currently, a comprehensive comparison of the relationship between individual lifestyle and CKD in a large-scale population is lacking.

Traditional approaches are difficult to analyze large datasets with multidimensional variables and non-linear relationships among risk factors and outcomes (23, 24). As a common form of artificial intelligence, machine learning successfully predicted long-term outcomes and selected suitable interventions, mainly depending on its high efficiency in handling tremendous variables and capturing high-dimensional relationships (24–28). Harnessing the cohort of UK biobank and supercomputer platform, we aimed to establish a machine learning-based CKD lifestyle-recommendation system, and test its performance *via* predicting the incidence of CKD.

## Methods

This study was an analysis of the UK Biobank cohort, which received approval from the National Information Governance Board for Health and Social Care and the National Health Service North West Multicenter Research Ethics Committee. All participants provided informed consent through electronic signature at the baseline assessment (29). The current analysis was approved by the UK Biobank with an ID of 66,536.

### Study population

The UK Biobank cohort recruited more than 500,000 community dwelling participants, aged 40–69 years, from across the United Kingdom. At baseline, we excluded participants with estimated glomerular filtration rate (eGFR) ≤ 60 ml/min/1.73 m^2^, chronic renal failure [International Classification of Diseases-10 (ICD-10) code: N18; or ICD-9 code: 5,859], self-reported chronic renal failure (code: 1,192), initiated renal replacement therapy (30), and subjects with <5% of whole lifestyle data.

### Assessment of lifestyle behaviors

We included diet, physical activity, smoking, alcohol drinking, sleep, and psychological health as the main six lifestyle behaviors (3, 31). For the assessment of dietary intakes, participants completed a questionnaire which included diet items with the frequency of consumption. Fruit, vegetable, bread, cereal, and muesli were recorded in slices per week, table spoons per day, pieces per day, and bowls per week (integer units). Intakes of meat, fish, and cheese responses were recorded as “less than once a week,” “once a week,” “two to four times a week,” “five or six times a week,” “once or more daily,” or “never” (polytomous variables). Tea or water intake was recorded in integer units of cups/glasses per day. We further categorized bread as white, brown, and whole-grain bread; cereal as bran, biscuit, oat cereal, or muesli; vegetable as raw and cooked vegetable; meat as pork, beef, and lamb; fish as oily fish and non-oily fish. The frequency of alcohol intake was recorded as “daily or almost daily,” “three or four times a week,” “one or two times a week,” “special occasions only or never” (polytomous variables). Smoking status and alcohol drinking status were categorized into current and non-current (such as never and former) at the time of assessment. An adequate sleep was defined as having a sleep duration of 7–9 h/day. We defined psychological health as participants who did not suffer from illness, injury, bereavement, and stress in the past 2 years.

For physical activity, participants were asked with “in the last 4 weeks did you spend any time doing the following: walking for pleasure, other sports (swimming/cycling/keeping fit), strenuous sports (requires sweat or hard breathing), light do it yourself (DIY) (e.g., pruning and watering the lawn) and heavy DIY (e.g., weeding, lawn mowing, carpentry, and digging).” In addition, we included variables of “frequency of stair climbing in the last 4 weeks (ranged from none to ≥20 times a day).” Time spent in vigorous, moderate activity, and walking was weighted by the energy expended for these categories of activity, to produce metabolic equivalent of task (MET) min/week of physical activity (calculation procedure of MET were provided in supplements). Vigorous activities included running, cycling uphill, carrying heavy furniture upstairs, martial arts, competitive sports, or intensive exercise. Moderate activities included walking upstairs, going the gym, jogging, energetic dancing aerobics, general sports, using heavy power tools, and other physically demanding DIY and gardening. For non-physical activities time, participants were asked with “in a typical day, how many hours do you spend on driving,” “how many hours do you spend using the computer,” and “how many hours do you spend watching the television.” Details on other covariates are described in the supplement.

### Outcomes

We used the equation of the Modification of Diet in Renal Disease study (MDRD) to calculate the eGFR. We defined CKD event incidence as one of the following conditions: eGFR ≤ 60 ml/min/1·73 m^2^ during the follow-up, mortality and hospitalization due to chronic renal failure and self-reported chronic renal failure (N18 of ICD-10 or 5,859 of ICD-9; or 1,192 of self-report code), initiation of renal replacement therapy (Z99.2,Z94.0 and Z49 of ICD-10) (30). Secondary outcomes included cardiovascular diseases and all-cause mortality.

### Statistical analysis

We implemented the model training and parameter tuning in machine learning with Python 3.8.3. Cox proportional hazards models and other statistical analyses were conducted using R 4.0.3. We conducted all the statistical analyses on the supercomputer platform (inspur M5).

#### Machine learning

Participants who developed CKD events were grouped as incident CKD. We classified those who were free of CKD events as non-CKD. We used the baseline lifestyle factors in both groups to perform the procedure of machine learning. Taking the accuracy of varied machine learning methods, speed and memory consumption into account, we chose the light gradient boosting machine (LightGBM) algorithm for machine learning. LightGBM is a gradient boosting framework that uses tree-based learning algorithms. We conducted the whole procedure with LightGBM Python-package (https://lightgbm.readthedocs.io/en/latest/Python-Intro.html), and parameters setting were provided in the supplement. In ranking the importance of individual variables, we adopted the conditional importance to avoid bias generated from multiple variables with different scales and variable collinearity in our dataset. The mean decrease impurity (MDI) of LightGBM was used as a measure of feature importance.

#### Cox proportional hazards models

Cox proportional hazards models were used to validate the association of individual lifestyle factors with incident CKD events. The duration of follow-up was calculated as the time between the baseline and the first occurrence of main outcome for participants who developed CKD events, or the end of follow-up for the current data release for those who were free of CKD events. Age and gender were adjusted as confounders. R 4.0.3 was used for the Cox proportional hazards regression (package “survival”) and proportional subdistribution hazards regression (package “cmprsk”). We used the ggsurvplot function in the survminer package to plot the cumulative hazard function.

#### Lifestyle score

The lifestyle scoring system was established based on the combination of machine learning and the Cox proportional-hazards model. The hazard ratios (*HR*s) were used to identify a healthy or unhealthy lifestyle factor, and the modified MDI (MDI/1,000) was adopted as the lifestyle score for every factor. The total lifestyle score equals to the scores of healthy lifestyle factors minus the scores of unhealthy lifestyle factors. We used restricted cubic spline regression analysis to explore the relationship between total lifestyle score and the incident CKD event. Receiver operator characteristic (ROC) curves and C-statistics were adopted to evaluate the performance of the lifestyle scoring system.

## Results

Complete data were available for 470,778 participants who were free of CKD in the UK Biobank study. After a median of 11-year follow-up, there were 13,555 participants who developed CKD events and 457,223 participants who stayed free of CKD. The baseline characteristics of these two groups are summarized in Table 1.

**Table 1:** Baseline characteristics of participants who kept free of CKD and during follow-up.

	**Keep free of CKD events**	**Develop CKD events**
No. of Participants	457,223	13,555
Male (%)	53.91	50.87
Age (years)	57 (8)	62 (6)
Ethnic background		
White (%)	94.57	94.04
Mixed (%)	0.60	0.44
Asian (%)	1.96	2.33
Black (%)	1.63	2.32
Chinese (%)	0.33	0.18
History of diabetes (%)	8.05	28.85
History of hypertension (%)	27.74	69.92
Body mass index (kg/m^2^)	27.29 (4.73)	29.39 (5.38)
Systolic blood pressure (mmHg)	137.52 (18.44)	144.01 (19.88)
Diastolic blood pressure (mmHg)	82.25 (10.05)	83.06 (10.49)
Mean arterial blood pressure (mmHg)	100.67 (11.86)	103.38 (12.26)
Fasting blood glucose (mmol/L)	5.10 (1.18)	5.62 (2.13)
Glycated hemoglobin (%)	5.43 (2.75)	5.44 (3.17)
Estimated GFR (ml/min/1.73 m^2^)	85.63 (14.97)	76.24 (14.48)
Urea nitrogen (mmol/L)	5.28 (1.22)	5.86 (1.39)
Uric acid (μmol/L)	305.11 (78.21)	335.34 (82.02)
Total cholesterol (mmol/L)	5.71 (1.13)	5.44 (1.26)
Triglycerides (mmol/L)	1.73 (1.02)	1.98 (1.12)
HDL cholesterol (mmol/L)	1.46 (0.38)	1.35 (0.37)
LDL cholesterol (mmol/L)	3.57 (0.86)	3.39 (0.94)
Current Smoking (%)	10.74	11.60
Current Alcohol Consumption (%)	92.22	87.78

*Data are represented as median (interquartile range). The glomerular filtration rate (GFR) was estimated with the use of the Modification of Diet in Renal Disease equation. CKD, chronic kidney disease; GFR, glomerular filtration rate; HDL, high density lipoprotein; LDL, low density lipoprotein*.

The importance of 37 lifestyle factors on incident CKD event was ordinally presented in Supplementary Figure 1. Bread, MET minutes of walking, MET minutes of moderate physical activity, tea, MET minutes of vigorous physical activity, water, cereal, raw vegetable, cooked vegetable, and fresh fruit ranked the top ten lifestyle factors associated with CKD.

In the age and gender adjusted Cox proportional hazards models, higher intakes of following dietary factors were associated with the lower risk of CKD event: whole grain bread (*HR* 0.77, 95% confidence interval (*CI*) 0.74–0.79), oat cereal (0.92, 0.89–0.96), muesli (0.74, 0.71–0.77), raw vegetable (0.99, 0.98–1.00), cooked vegetable (0.98, 0.97–0.99), fresh fruit (0.98, 0.97–0.99), dried fruit (0.68, 0.62–0.74), cheese (0.56, 0.49–0.65 for one time or more daily), oily fish (0.72, 0.68–0.77 for two times or more in a week), and non-oily fish (0.87, 0.79–0.95 for two times or more in a week); while higher intakes of following dietary factors were associated with the higher risk of CKD event: white bread (1.36, 1.31–1.41), biscuit cereal (1.17, 1.12–1.22), processed meat (1.40, 1.31–1.50 for two times or more in a week), salt added to food (1.17, 1.08–1.26 for always), pork (1.20, 1.12–1.30 for two times or more in a week), poultry (1.29, 1.18–1.42 for two times or more in a week), beef (1.46, 1.34–1.60 for two times or more in a week), and lamb (1.23, 1.12–1.35 for two times more in a week). For physical activity, walking MET >2,000 min/week (0.94, 0.90–0.99), moderate PA MET >800 min/week (0.96, 0.93–1.00), climbing stair (0.62, 0.57–0.68 for more than 20 times per day), usual walking pace (0.33, 0.32–0.35 at brisk pace), walk for pleasure (0.69, 0.67–0.72), swimming/cycling/keeping fit (0.72, 0.70–0.75), light DIY (0.65, 0.60–0.70), heavy DIY (0.86, 0.83–0.89), and strenuous sports (0.78, 0.75–0.81) were associated with the lower risk of CKD event. In addition, the longer time of mild to moderate physical activity did the subjects reported, the lower the risk of CKD they had. Subjects with current smoking (1.28, 1.21–1.35), tea >5 cups/day (1.12, 1.07–1.18), lower drinking temperature (1.06, 1.00–1.13), variation in diet (1.33, 1.25–1.41), and psychological stress (1.27, 1.23–1.31) showed the higher risk of CKD event, while current alcohol drinking (0.66, 0.62–0.69) and adequate sleep (0.81, 0.78–0.84) were associated with a lower risk (Supplementary Figure 2).

**Figure 1:**
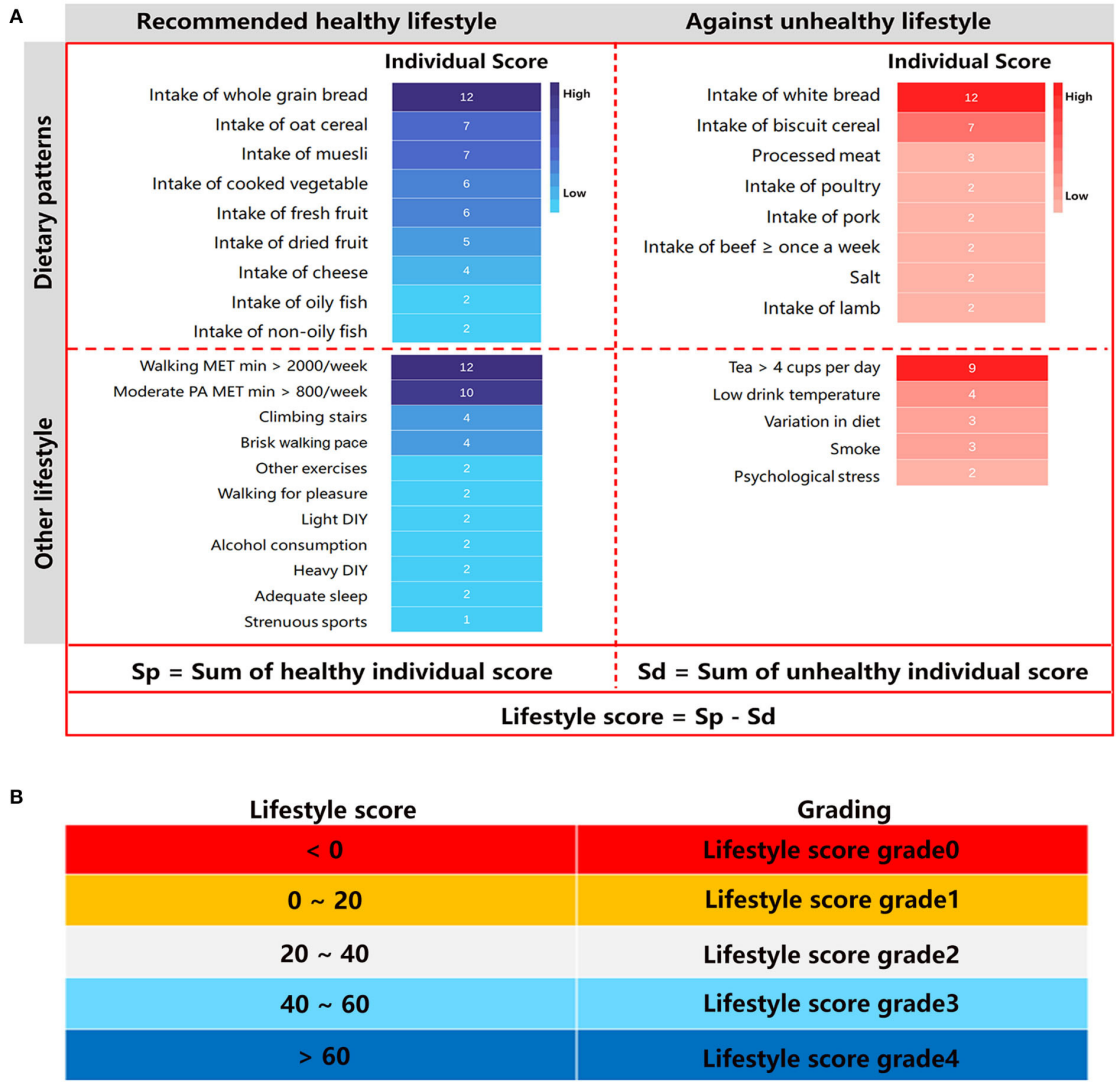
Score of lifestyle factors. **(A)** Healthy and unhealthy lifestyles are categorized according to the hazard ratios (*HR*s) in Supplementary Figure 2. The mean decrease impurity (MDI)/1,000 was adopted as the lifestyle score for every factor. Moderate PA included walking upstairs, going the gym, jogging, energetic dancing aerobics, most sports, using heavy power tools, and other physically demanding DIY and gardening. Light DIY included pruning, watering the lawn; other exercises included swimming, cycling, keeping fit, and bowling; Heavy DIY included weeding, lawn mowing, carpentry, and digging. PA, physical activity; DIY, do-it-yourself; MET, Metabolic Equivalent Task. **(B)** The lifestyle score was categorized as <0, 0–20, 20–40, 40–60, corresponding to grade 0, grade 1, grade 2, grade 3 and grade 4 respectively.

**Figure 2:**
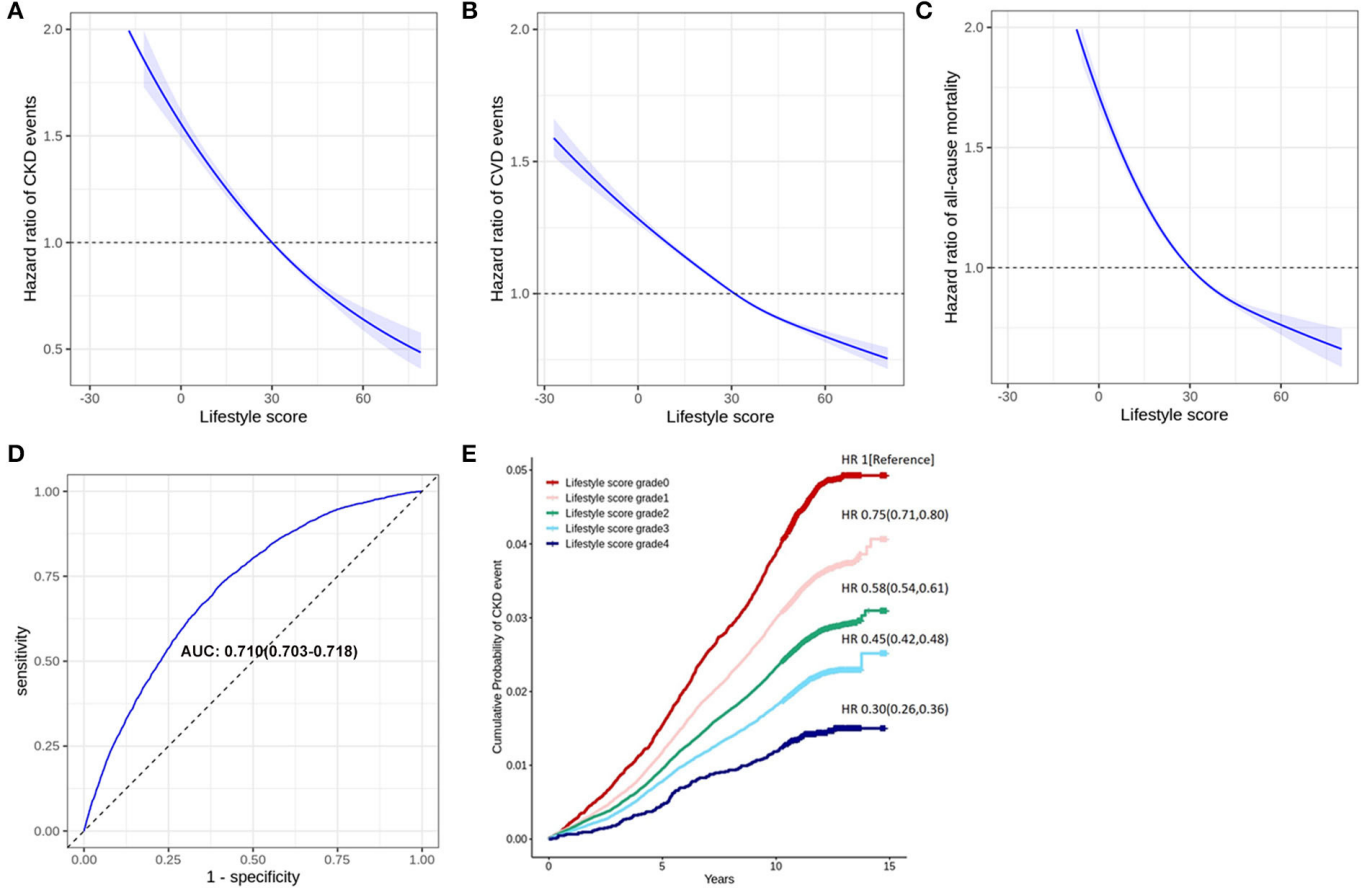
Validation of the lifestyle score in long-term outcomes. Panel **(A)** shows a restricted cubic spline regression analysis, which indicates a linear relationship between the total lifestyle score (equals to the scores of healthy lifestyle factors minus the scores of unhealthy lifestyle factors) and risk of CKD events. Panel **(B)** shows the categorization for risk of CKD event according to the total lifestyle score. Panel **(C)** shows the receiver operator characteristic curves (ROC) of the age-adjusted lifestyle score. Panel **(D)** or **(E)** is a restricted cubic spline regression analysis, which indicates a linear relationship between the total lifestyle score (equals to the scores of healthy lifestyle factors minus the scores of unhealthy lifestyle factors) and the risk of CVD events or all-cause mortality.

Adopting the modified MDI (MDI/1,000) as the individual lifestyle score for healthy and unhealthy lifestyle factors, we summarized the lifestyle score in Figure 1. As the restricted cubic spline regression analysis showed, the relationship between the lifestyle score and the risk of CKD event was manifested as linear (*p* for linear <0.001) (Figure 2A). A higher individual lifestyle score was significantly associated with a lower risk of cardiovascular diseases (Figure 2B) or all-cause mortality (Figure 2C). Compared with participants with lifestyle score <0, participants scoring 0–20, 20–40, 40–60, and >60 exhibited 25% (20%, 29%), 42% (39%, 46%), 55% (52%, 58%) and 70% (64%, 74%) lower risk of CKD event, respectively (Figure 2E). For predicting CKD events by the age-adjusted lifestyle score, the area under the curve (AUC) of ROC was 0.710 (0.703, 0.718) and C-statistics 0.706 (0.704, 0.708) (Figure 2D).

## Discussion

With a systematic comparison of 37 lifestyle factors effects on CKD risk by machine learning, we established a lifestyle scoring system for CKD risk identification. The relationship between the lifestyle score and the risk of CKD event was linear, and an ROC analysis proved that the system was effective in predicting the CKD incidence. Our study not only confirmed that the majority of CVD-based healthy lifestyle factors were applicable to the prediction of CKD, but also, for the first time, provided unified hierarchies of evidence for preventing CKD by lifestyle.

Recent studies involved in the relationship between diet and CKD have exhibited inconsistent results. In some cohort studies, healthy dietary patterns such as Dietary Approaches to Stop Hypertension (DASH) and multicomponent diet were shown to be associated with the lower risk of CKD incidence (32, 33). However, other cohort studies indicated that a healthy dietary pattern (DASH or American Heart Association recommended dietary pattern) was not associated with the incident CKD (11, 12). Using a large-scale cohort of UK Biobank, we confirmed that whole-grain bread, oat cereal, muesli, cheese, fruit, vegetable, and fish were associated with the lower risk of CKD incidence, supporting that healthy dietary patterns are beneficial in CKD prevention. Previous studies suggested that both low and high intake of water were not beneficial in CKD (34), while no significant association was observed between tea and the risk of CKD (35, 36). In our dataset, an excessive intake of tea (>5 cups per day) or water (>4 glasses per day) was associated with the higher risk of developing CKD, which argued for an appropriate consumption of tea or water.

Previous studies suggested that a higher level of total physical activity was associated with a lower risk of developing CKD or ESRD (13, 37), which were also validated in our study. Beyond total physical activity, our analysis of individual physical activity showed that spending any time for walking, swimming/cycling/keeping fit, stair climbing, and light/heavy DIY were associated with the lower risk of CKD event, indicating that occasional light physical activity was also helpful in the prevention of CKD. In addition, a meta-analysis showed that both short- and long-sleep durations were associated with a higher risk of CKD (20), and psychological distress was also shown to be positively associated with the CKD risk (38). Both sleep and psychological health were validated to be the predictors of CKD event in our analysis.

Previous studies on the relationship between alcohol intake and the risk of CKD were inconsistent. A cohort study showed that regular and occasional binge alcohol drinking were associated with a higher risk of CKD progression when compared with non-drinking (15). In contrast, a meta-analysis indicated that compared to participants with a minimum alcohol consumption, those who had light to heavy (>12 g/day) intake of alcohol were associated with a lower risk of CKD (16). In our study, although the importance of alcohol consumption on CKD was not as high as diet or physical activity, a higher intake of alcohol was associated with a lower risk of CKD, arguing a protective effect of alcohol consumption on the CKD progression. It was suggested that a higher level of high-density lipoprotein cholesterol (HDL-C) and a lower level of plasminogen activator inhibitor-1 might explain the protective role of alcohol (16). Apart from alcohol, our results also demonstrated the detrimental effect of smoking on CKD, which is similar to previous findings (18).

The main strengths of this study included a large-scale population with more than 450,000 participants, long follow-up time, and a comprehensive lifestyle evaluation. More importantly, we used supercomputer to analyze multidimensional variables and non-linear relationships by machine learning, which might be more efficient and accurate than traditional methods. Our study also had some limitations. On one hand, most lifestyle factors were evaluated only at baseline. Although the large-scale population diminished the impact of one-time evaluation bias, repeat assessments often leads to more convincing results. On the other hand, most participants adopted the western lifestyle, which is different from Asia, Africa, and other areas. Whether the lifestyle scoring works well in different populations needs to be further verified. In addition, more than 90% of our participants were Whites. There are significant ethnic disparities in CKD progression (39), and the influence of lifestyle factors on CKD was shown to be varied among different ethnicities (40). Although the homogeneity of race is helpful for controlling confounders, our findings need to be replicated in other populations, such as Asians and blacks.

## Conclusion

In conclusion, a lifestyle scoring system for CKD prevention established by integrating dietary patterns, physical activity, sleep, psychological health, smoking, and alcohol was associated with an increased risk of CKD. On the basis of the lifestyle scoring system for CKD risk, people could flexibly choose healthy lifestyles and avoid unhealthy lifestyles. Further study is needed to verify whether the system will improve clinical care and patient outcomes.

### Practical application

Harnessing the cohort of UK biobank and supercomputer platform, we established a lifestyle scoring system for CKD prevention. Based on the system, individuals could flexibly choose healthy lifestyles and avoid unhealthy lifestyles to prevent CKD.

## Data availability statement

The raw data supporting the conclusions of this article will be made available by the authors, without undue reservation.

## Ethics statement

The studies involving human participants were reviewed and approved by National Information Governance Board for Health and Social Care and the National Health Service North West Multicenter Research Ethics Committee. The patients/participants provided their written informed consent to participate in this study.

## Author contributions

JH, SY, ZW, and QL designed the study, oversaw the data analysis, and interpreted the data. JH and WL conducted the data analysis and wrote the manuscript. XC contributed to the data analysis and writing of the manuscript. RG and ZL contributed to the study conception and design. BP provided statistical expertise. YW, TL, and YY assisted with the data analysis. LM, MM, and LG contributed to the writing and editing of the manuscript. SY, ZW, and JH are the guarantors of this work and, as such, had full access to all the data in the study and takes responsibility for the integrity of the data and the accuracy of the data analysis. All authors contributed to the article and approved the submitted version.

## Funding

This work was supported by the National Natural Science Foundation of China (81870567, 81800731, 81970720, and 81800757), the National Key Research and Development plan, Major Project of Precision Medicine Research (2017YFC0909600, sub-project: 2017YFC0909602, and 2017YFC0909603), the Bethune Merck Diabetes Research Foundation (G2018030), the Technological Innovation and Application Development Project of Chongqing (cstc2019jscx-msxmX0207), the Chongqing Science and Health Joint Medical Research Project (2020FYYX141), the China International Medical Foundation (Z-2017-26-1902-2), the High-end Medical Talents of Middle-aged and Young People in Chongqing [yuweiren (2015) 49], the Yong and Middle-aged Senior Medical Talents studio of Chongqing (ZQNYXGDRCGZS2021001), the Chongqing Outstanding Youth Funds (cstc2019jcyjjq0006), and the Outstanding Talents of the First Affiliated Hospital of Chongqing Medical University 2019 (to JH, 2019-4-22).

## Conflict of interest

The authors declare that the research was conducted in the absence of any commercial or financial relationships that could be construed as a potential conflict of interest.

## Publisher's note

All claims expressed in this article are solely those of the authors and do not necessarily represent those of their affiliated organizations, or those of the publisher, the editors and the reviewers. Any product that may be evaluated in this article, or claim that may be made by its manufacturer, is not guaranteed or endorsed by the publisher.

## References

[1] Kalantar-Zadeh K Jafar TH Nitsch D Neuen BL Perkovic V. Chronic kidney disease. Lancet. (2021) 398:786–802. 10.1016/S0140-6736(21)00519-5 34175022

[2] GBD Chronic Kidney Disease Collaboration. Global, regional, and national burden of chronic kidney disease, 1990-2017: a systematic analysis for the global burden of disease study 2017. Lancet. (2020) 395:709–33. 10.1016/S0140-6736(20)30045-3 32061315 PMC7049905

[3] Li Y Schoufour J Wang DD Dhana K Pan A Liu X. Healthy lifestyle and life expectancy free of cancer, cardiovascular disease, and type 2 diabetes: prospective cohort study. BMJ. (2020) 368:l6669. 10.1136/bmj.l6669 31915124 PMC7190036

[4] Benjamin EJ Blaha MJ Chiuve SE Cushman M Das SR Deo R. Heart disease and stroke statistics-2017 update: a report from the American heart association. Circulation. (2017) 135:e146–603. 10.1161/CIR.0000000000000491 28122885 PMC5408160

[5] Miller V Mente A Dehghan M Rangarajan S Zhang X Swaminathan S. Fruit, vegetable, and legume intake, and cardiovascular disease and deaths in 18 countries (PURE): a prospective cohort study. Lancet. (2017) 390:2037–49. 10.1016/S0140-6736(17)32253-5 28864331

[6] Shan Z Li Y Baden MY Bhupathiraju SN Wang DD Sun Q. Association between healthy eating patterns and risk of cardiovascular disease. JAMA Intern Med. (2020) 180:1090–100. 10.1001/jamainternmed.2020.2176 32539102 PMC7296454

[7] Sotos-Prieto M Bhupathiraju SN Mattei J Fung TT Li Y Pan A. Association of changes in diet quality with total and cause-specific mortality. N Engl J Med. (2017) 377:143–53. 10.1056/NEJMoa1613502 28700845 PMC5589446

[8] Yates T Haffner SM Schulte PJ Thomas L Huffman KM Bales CW. Association between change in daily ambulatory activity and cardiovascular events in people with impaired glucose tolerance (NAVIGATOR trial): a cohort analysis. Lancet. (2014) 383:1059–66. 10.1016/S0140-6736(13)62061-9 24361242

[9] Gansevoort RT Correa-Rotter R Hemmelgarn BR Jafar TH Heerspink HJ Mann JF. Chronic kidney disease and cardiovascular risk: epidemiology, mechanisms, and prevention. Lancet. (2013) 382:339–52. 10.1016/S0140-6736(13)60595-4 23727170

[10] Bach KE Kelly JT Palmer SC Khalesi S Strippoli GFM Campbell KL. Healthy dietary patterns and incidence of CKD: a meta-analysis of cohort studies. Clin J Am Soc Nephrol. (2019) 14:1441–9. 10.2215/CJN.00530119 31551237 PMC6777603

[11] Rebholz CM Anderson CA Grams ME Bazzano LA Crews DC Chang AR. Relationship of the American heart association's impact goals (Life's Simple 7) With risk of chronic kidney disease: results from the Atherosclerosis Risk in Communities (ARIC) cohort study. J Am Heart Assoc. (2016) 5:e003192. 10.1161/JAHA.116.003192 27053058 PMC4859292

[12] Liu Y Kuczmarski MF Miller ER 3rd Nava MB Zonderman AB Evans MK. Dietary habits and risk of kidney function decline in an urban population. J Ren Nutr. (2017) 27:16–25. 10.1053/j.jrn.2016.08.007 27771303 PMC5161560

[13] Guo C Tam T Bo Y Chang LY Lao XQ Thomas GN. Habitual physical activity, renal function and chronic kidney disease: a cohort study of nearly 200,000 adults. Br J Sports Med. (2020) 54:1225–30. 10.1136/bjsports-2019-100989 31969348

[14] Yamamoto S Inoue Y Kuwahara K Miki T Nakagawa T Honda T. Leisure-time, occupational, and commuting physical activity and the risk of chronic kidney disease in a working population. Sci Rep. (2021) 11:12308. 10.1038/s41598-021-91525-4 34112832 PMC8192894

[15] Joo YS Koh H Nam KH Lee S Kim J Lee C. Alcohol consumption and progression of chronic kidney disease: results from the korean cohort study for outcome in patients with chronic kidney disease. Mayo Clin Proc. (2020) 95:293–305. 10.1016/j.mayocp.2019.06.014 31883696

[16] Yuan HC Yu QT Bai H Xu HZ Gu P Chen LY. Alcohol intake and the risk of chronic kidney disease: results from a systematic review and dose-response meta-analysis. Eur J Clin Nutr. (2021) 75:1555–67. 10.1038/s41430-021-00873-x 33674776

[17] Koning SH Gansevoort RT Mukamal KJ Rimm EB Bakker SJ Joosten MM. Alcohol consumption is inversely associated with the risk of developing chronic kidney disease. Kidney Int. (2015) 87:1009–16. 10.1038/ki.2014.414 25587707

[18] Xia J Wang L Ma Z Zhong L Wang Y Gao Y. Cigarette smoking and chronic kidney disease in the general population: a systematic review and meta-analysis of prospective cohort studies. Nephrol Dial Transplant. (2017) 32:475–87. 10.1093/ndt/gfw452 28339863

[19] Franceschini N Deng Y Flessner MF Eckfeldt JH Kramer HJ Lash JP. Smoking patterns and chronic kidney disease in US Hispanics: Hispanic community health study/study of Latinos. Nephrol Dial Transplant. (2016) 31:1670–6. 10.1093/ndt/gfw210 27257272 PMC5039342

[20] Hao Q Xie M Zhu L Dou Y Dai M Wu Y. Association of sleep duration with chronic kidney disease and proteinuria in adults: a systematic review and dose-response meta-analysis. Int Urol Nephrol. (2020) 52:1305–20. 10.1007/s11255-020-02488-w 32418007

[21] Knowles S Swan L Salzberg M Castle D Langham R. Exploring the relationships between health status, illness perceptions, coping strategies and psychological morbidity in a chronic kidney disease cohort. Am J Med Sci. (2014) 348:271–6. 10.1097/MAJ.0000000000000242 24751421

[22] Choi NG Sullivan JE DiNitto DM Kunik ME. Associations between psychological distress and health-related behaviors among adults with chronic kidney disease. Prev Med. (2019) 126:105749. 10.1016/j.ypmed.2019.06.007 31199950

[23] Schwalbe N Wahl B. Artificial intelligence and the future of global health. Lancet. (2020) 395:1579–86. 10.1016/S0140-6736(20)30226-9 32416782 PMC7255280

[24] D'Ascenzo F De Filippo O Gallone G Mittone G Deriu MA Iannaccone M. Machine learning-based prediction of adverse events following an acute coronary syndrome (PRAISE): a modelling study of pooled datasets. Lancet. (2021) 397:199–207. 10.1016/S0140-6736(20)32519-8 33453782

[25] Motwani M Dey D Berman DS Germano G Achenbach S Al-Mallah MH. Machine learning for prediction of all-cause mortality in patients with suspected coronary artery disease: a 5-year multicentre prospective registry analysis. Eur Heart J. (2017) 38:500–7. 10.1093/eurheartj/ehw188 27252451 PMC5897836

[26] Tokodi M Schwertner WR Kovács A Tosér Z Staub L Sárkány A. Machine learning-based mortality prediction of patients undergoing cardiac resynchronization therapy: the SEMMELWEIS-CRT score. Eur Heart J. (2020) 41:1747–56. 10.1093/eurheartj/ehz902 31923316 PMC7205468

[27] Yelin I Snitser O Novich G Katz R Tal O Parizade M. Personal clinical history predicts antibiotic resistance of urinary tract infections. Nat Med. (2019) 25:1143–52. 10.1038/s41591-019-0503-6 31273328 PMC6962525

[28] Didelot X Pouwels KB. Machine-learning-assisted selection of antibiotic prescription. Nat Med. (2019) 25:1033–4. 10.1038/s41591-019-0517-0 31273329

[29] Pilling LC Tamosauskaite J Jones G Wood AR Jones L Kuo CL. Common conditions associated with hereditary haemochromatosis genetic variants: cohort study in UK Biobank. BMJ. (2019) 364:k5222. 10.1136/bmj.k5222 30651232 PMC6334179

[30] Zhao JV Schooling CM. Sex-specific associations of sex hormone binding globulin with CKD and Kidney function: a univariable and multivariable mendelian randomization study in the UK Biobank. J Am Soc Nephrol. (2021) 32:686–94. 10.1681/ASN.2020050659 33318152 PMC7920164

[31] Kris-Etherton PM Petersen KS Després JP Braun L de Ferranti SD Furie KL. Special considerations for healthy lifestyle promotion across the life span in clinical settings: a science advisory from the American heart association. Circulation. (2021) 144:e515–32. 10.1161/CIR.0000000000001014 34689570

[32] Dunkler D Kohl M Teo KK Heinze G Dehghan M Clase CM. Population-attributable fractions of modifiable lifestyle factors for CKD and mortality in individuals with type 2 diabetes: a cohort study. Am J Kidney Dis. (2016) 68:29–40. 10.1053/j.ajkd.2015.12.019 26830448

[33] Asghari G Yuzbashian E Mirmiran P Azizi F. The association between dietary approaches to stop hypertension and incidence of chronic kidney disease in adults: the tehran lipid and glucose study. Nephrol Dial Transplant. (2017) 32:ii224–30. 10.1093/ndt/gfw273 28201810

[34] Wagner S Merkling T Metzger M Bankir L Laville M Frimat L. Water intake and progression of chronic kidney disease: the CKD-REIN cohort study. Nephrol Dial Transplant. (2021) 37:730–9. 10.1093/ndt/gfab036 33576809

[35] Herber-Gast GC van Essen H Verschuren WM Stehouwer CD Gansevoort RT Bakker SJ. Coffee and tea consumption in relation to estimated glomerular filtration rate: results from the population-based longitudinal doetinchem cohort study. Am J Clin Nutr. (2016) 103:1370–7. 10.3945/ajcn.115.112755 26984487

[36] Gaeini Z Bahadoran Z Mirmiran P Azizi F. Tea, coffee, caffeine intake and the risk of cardio-metabolic outcomes: findings from a population with low coffee and high tea consumption. Nutr Metab. (2019) 16:28. 10.1186/s12986-019-0355-6 31073321 PMC6500051

[37] Kuo CP Tsai MT Lee KH Lin YP Huang SS Huang CC. Dose-response effects of physical activity on all-cause mortality and major cardiorenal outcomes in chronic kidney disease. Eur J Prev Cardiol. (2021) 29:452–61. 10.1093/eurjpc/zwaa162 33704426

[38] Zalai D Szeifert L Novak M. Psychological distress and depression in patients with chronic kidney disease. Semin Dial. (2012) 25:428–38. 10.1111/j.1525-139X.2012.01100.x 22809005

[39] Chu CD Powe NR McCulloch CE Crews DC Han Y Bragg-Gresham JL. Trends in chronic kidney disease care in the US by race and ethnicity, 2012–2019. JAMA Netw Open. (2021) 4:e2127014. 10.1001/jamanetworkopen.2021.27014 34570204 PMC8477264

[40] Crews DC Banerjee T Wesson DE Morgenstern H Saran R Burrows NR. Race/ethnicity, dietary acid load, and risk of end-stage renal disease among US adults with chronic kidney disease. Am J Nephrol. (2018) 47:174–81. 10.1159/000487715 29525790 PMC5906156

