# Supplementary material for: Lifestyle and chronic kidney disease: A machine learning modeling study
Source: Front Nutr. 2022 Jul 22;9:918576. doi: 10.3389/fnut.2022.918576 (PMC9355159; doi:10.3389/fnut.2022.918576)
Supplement: Supplementary file 1 [file Data_Sheet_1.docx]

**Supplements**

**Lifestyle and Chronic Kidney Disease:** **A Machine Learning Modeling Study**

Running title: Lifestyle and CKD

Wenjin Luo^1^, Xiangjun Chen^1^, Rufei Gao^2^, Bin Peng^3^, Yue Wang^1^, Ting Luo^1^, Yi Yang^1^, Bing Kang^4^, Chuan Peng^5^, Linqiang Ma^1^, Mei Mei^1^, Lilin Gong^1^, Zhiping Liu^1^, Qifu Li^1^, Shumin Yang^1^, Zhihong Wang^1#^, Jinbo Hu^1#^

**Affiliations:**

^1^ Department of Endocrinology, the First Affiliated Hospital of Chongqing Medical University, Chongqing, China.

^2^ Laboratory of Reproductive Biology, School of Public Health and Management, Chongqing Medical University, Chongqing, China.

^3^ School of Public Health and Management, Chongqing Medical University, Chongqing, China.

^4^ Department of Clinical Nutrition, the First Affiliated Hospital of Chongqing Medical University, Chongqing, China.

^5^ The Chongqing Key Laboratory of Translational Medicine in Major Metabolic Diseases, the First Affiliated Hospital of Chongqing Medical University, Chongqing, China.

^#^ Corresponding author and person to whom reprint requests should be addressed:

Jinbo Hu (hujinbo_568@163.com), Zhihong Wang(e-mail:towzh713@126.com)

Department of Endocrinology, The First Affiliated Hospital of Chongqing Medical University

No.1 Youyi Street, Yuzhong District, Chongqing, China (400016). Phone: +86-023-89011552;

Fax: +86-023-89011552

**Methods**

**Metabolic equivalent (MET) of physical activity**

An average MET score was derived for each type of activity [^1^](#_ENREF_1)^,^[^2^](#_ENREF_2): walking = 3.3 METs, moderate physical activity = 4.0 METs and vigorous physical activity = 8.0 METs. Using these values, four continuous scores are defined: walking MET-minutes/week = 3.3 × walking minutes × walking days; moderate MET-minutes/week = 4.0 × moderate-intensity activity minutes × moderate days; vigorous MET-minutes/week = 8.0 × vigorous-intensity activity minutes × vigorous-intensity days.

**Covariates**

We defined diabetes as fasting glucose of ≥ 7.0 mmol/L, nonfasting glucose ≥ 11.1 mmol/L HbA1c ≥ 6.5%, self-reported diabetes or use of antidiabetic medications. Hypertension was defined as blood pressure ≥ 140/90 mmHg or the use of antihypertensive drugs. We defined a history of cardiovascular disease as coronary heart disease, intermittent claudication, congestive heart failure, stroke or transient ischemic attack. Circulating concentrations of total cholesterol (TC), triglycerides (TG), high-density lipoprotein cholesterol (HDL-c), and low-density lipoprotein cholesterol (LDL-c) were measured according to the biomarker assay quality procedures of UK Biobank.

**Data Imputation**

Missing data was imputed to maximize power and avoid selection bias. We used multivariate imputation by chained equations (MICE) to impute all the missing data. MICE is operated under the assumption that missing is completely at random. We imputed every missing variable with mean values, and temporarily set all missing values as the mean observed values. The imputed mean values were then set back to missing data. We established models of linear and logistic regression for all continuous and categorical variables, and used completed data to predict missing values. All missing values are subsequently replaced with coefficients generated from imputed models. These steps are repeated for all variables with missing values. Validation for imputed models are achieved by comparing distributions of raw data with imputed data.The estimates from each imputed dataset were merged into one overall estimate with the use of Rubin’s rule. For the main analysis, we pooled results across all imputed datasets.

**General description of Machine learning**

Participants who developed CKD event were grouped as incident CKD, and those who were free of CKD event during follow-up were grouped as non-CKD. We used the baseline lifestyle factors to perform the procedure of machine learning. Considering for the accuracy of varied machine learning methods, speed and memory consumption, we chose light gradient boosting machine (LightGBM) as the algorithm of machine learning.

LightGBM is a highly efficient gradient boosting decision tree (GBDT) implementation that can handle a large number of data instances and a large number of data features without influencing the accuracy and overfitting. As a nonparametric ensemble model from decision tree, LightGBM consists of bagging and boosting techniques to increase accuracy and robustness and reduce variance of predictions. The algorithm of LightGBM incorporates GOSS (Gradient-based One-Side Sampling) to split the optimal node through calculating variance gain, improves efficiency by distinguishing samples of varied gradients, retains samples of larger gradients and randomly samples smaller gradients to reduce the amount of computation. LightGBM reduces the feature dimension to a certain extent by means of Exclusive Feature Bundling, which speeds up the training process of GBDT via bundling many exclusive features to reduce feature dimensions. Beyond traditional level-wise algorithm, the leaf-wise algorithm of LightGBM diminishes more loss with better prediction accuracy, which can rarely be achieved by existing boosting algorithms.

In one word, the prominences of LightGBM included higher training efficiency, lower memory usage, better accuracy, and handling large-scale data.

**Procedures of LightGBM**

The dataset was split into training subset and testing subset (80% and 20%. respectively) for 10-fold cross-validation. The Bayesian optimization approach was used for hyperparameter tuning and the model architecture. We set up the following parameters in the tuning process: max_depth (controls the max depth of the trees) as 166, learning_rate (controls the rate of learning) as 0.09, num_leaves (controls the number of leaves) as 33, min_child_weight (controls the minimum sum of instance weight of all observations) as 0.004, subsample (controls the subsample ratio of the training instance) as 0.6, colsample_bytree (controls the subsample ratio of columns when constructing individual tree) as 0.9.

Procedures of LightGBM were performed in Python 3.8.3 and R 4.0.3 with bayesian-optimization 1.2.0, imbalanced-learn 0.8.0, lightgbm 3.2.1,matplotlib 3.1.1, numpy 1.19.2, pandas 1.2.3, scikit-learn 0.24.1, and data.table 1.14.0, MICE 3.13.0, tidyverse 1.3.0, survival 3.2-7, respectively.

**Performance of LightGBM**

The LightGBM learning curve which described the relation between training examples and score was presented as the following figure.


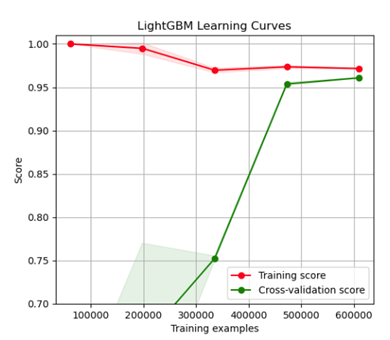


The scalability of the model which described the relation between training examples and fit times was presented as the following figure.


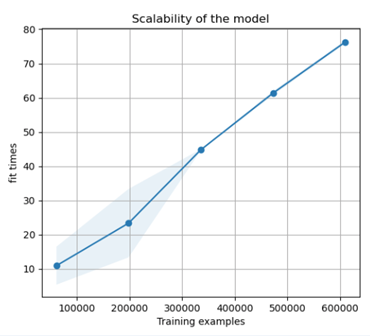


The performance of the model which described the relation between fit times and score was presented as the following figure.


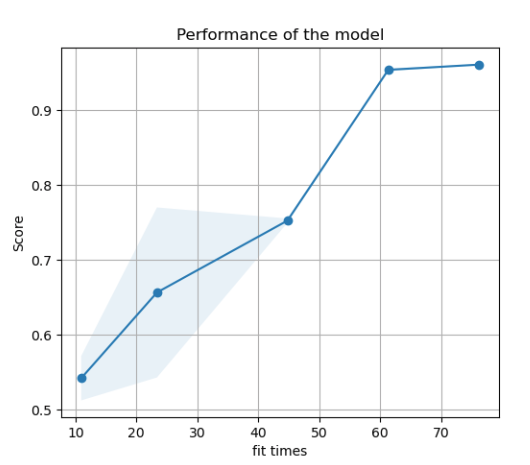


**Reference**

1. Ainsworth BE, Haskell WL, Whitt MC, et al. Compendium of physical activities: an update of activity codes and MET intensities. *Med. Sci. Sports Exerc.* Sep 2000;32(9 Suppl):S498-504.

2. Cassidy S, Chau JY, Catt M, Bauman A, Trenell MI. Cross-sectional study of diet, physical activity, television viewing and sleep duration in 233,110 adults from the UK Biobank; the behavioural phenotype of cardiovascular disease and type 2 diabetes. *BMJ open.* Mar 15 2016;6(3):e010038.


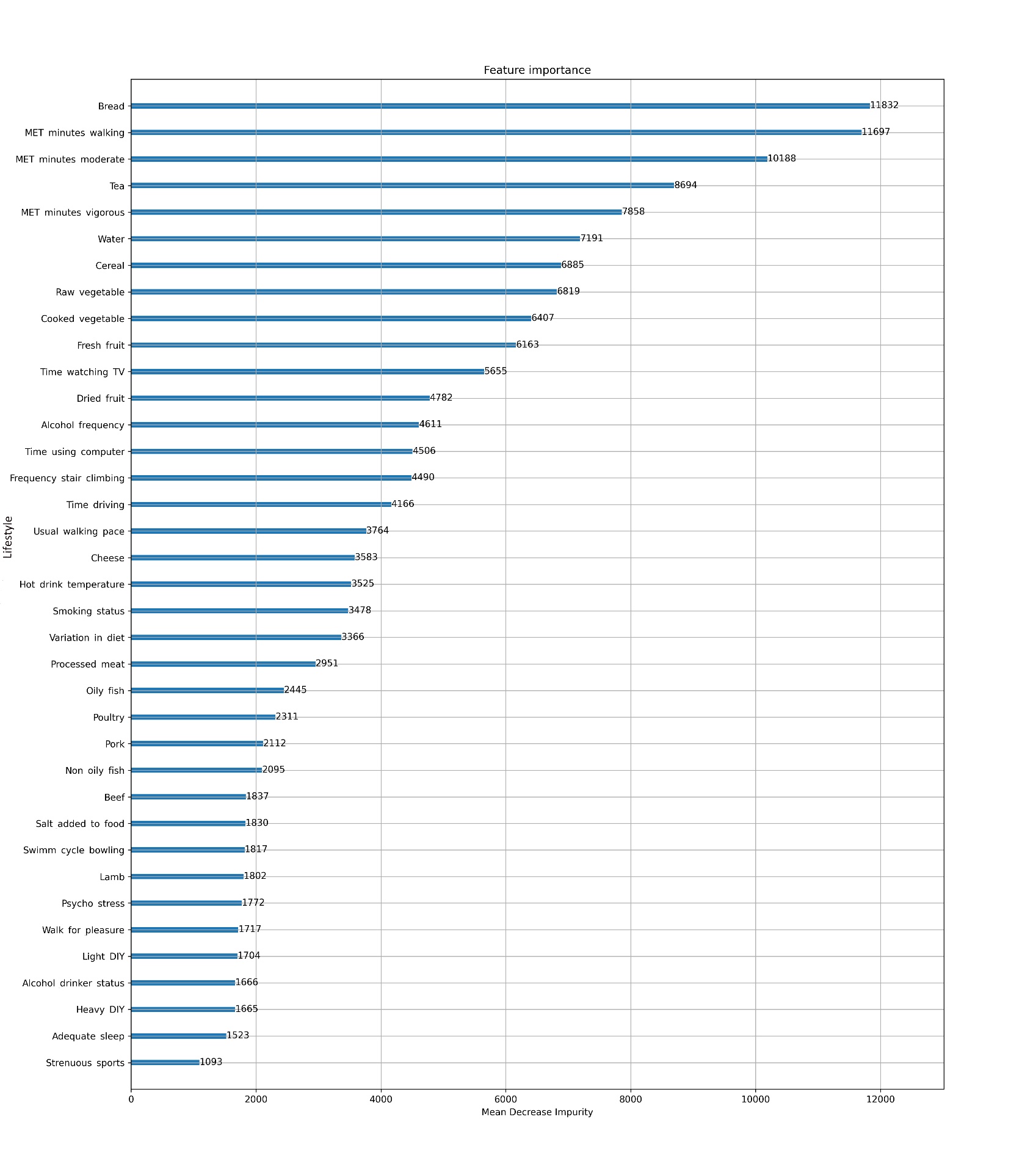


**Supplement figure 1. The importance of 37 lifestyle factors with CKD**

Machine learning based on light gradient boosting machine algorithm was used to rank the importance of 35 lifestyle factors. The mean decrease impurity (MDI) was used as a measure of feature importance.

Time spent in vigorous, moderate activity and walking was weighted by the energy expended for these categories of activity, to produce metabolic equivalent of task (MET) min/week of physical activity. Moderate PA (physical activity) included walking upstairs, going the gym, jogging, energetic dancing aerobics, general sports, using heavy power tools and other physically demanding DIY & gardening. Vigorous PA included running, cycling uphill, carrying heavy furniture upstairs, martial arts, competitive sports or intensive exercise. Light DIY included pruning, watering the lawn; other exercises included swimming, cycling, keeping fit and bowling; Heavy DIY included weeding, lawn mowing, carpentry and digging.


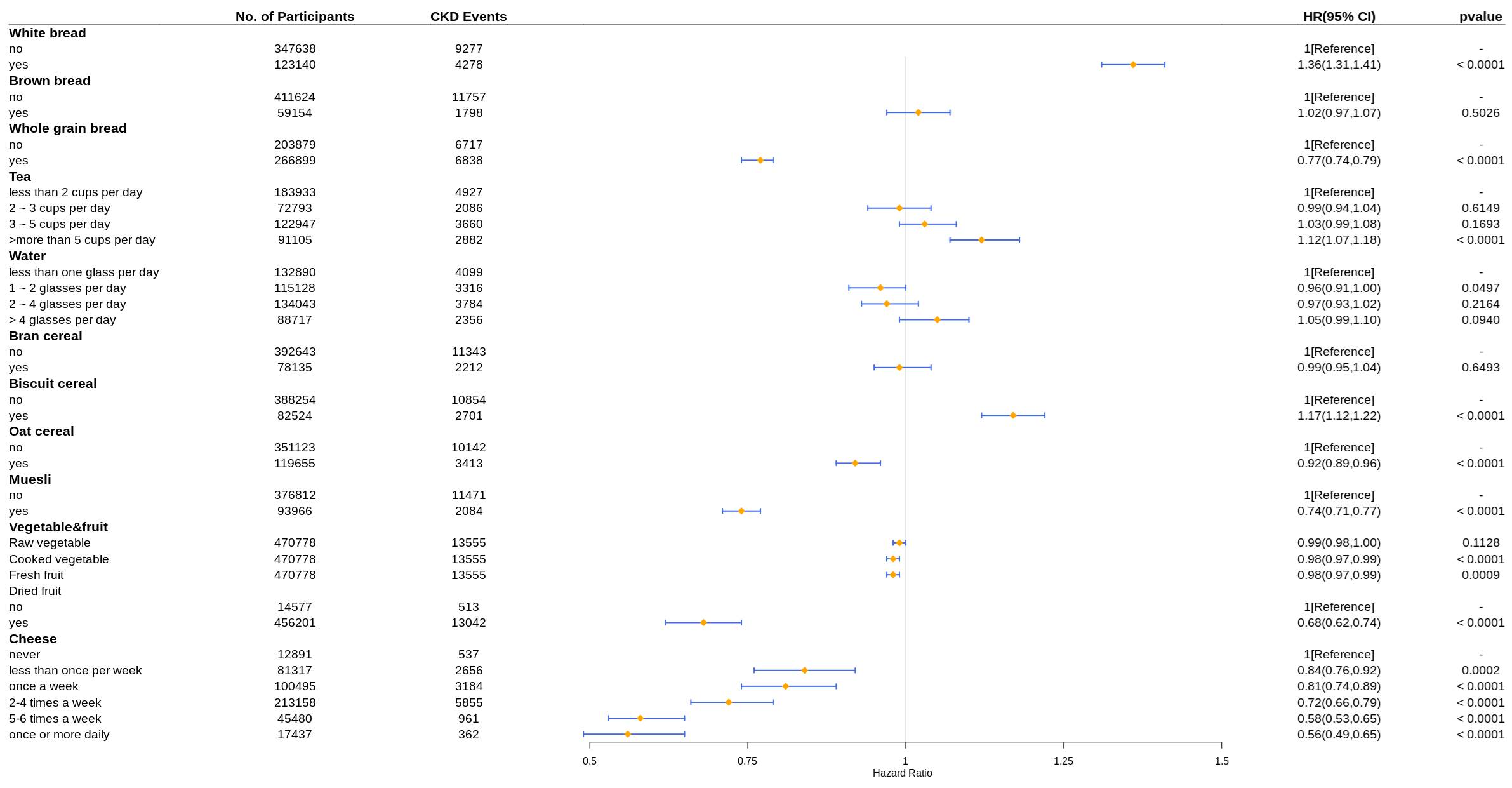


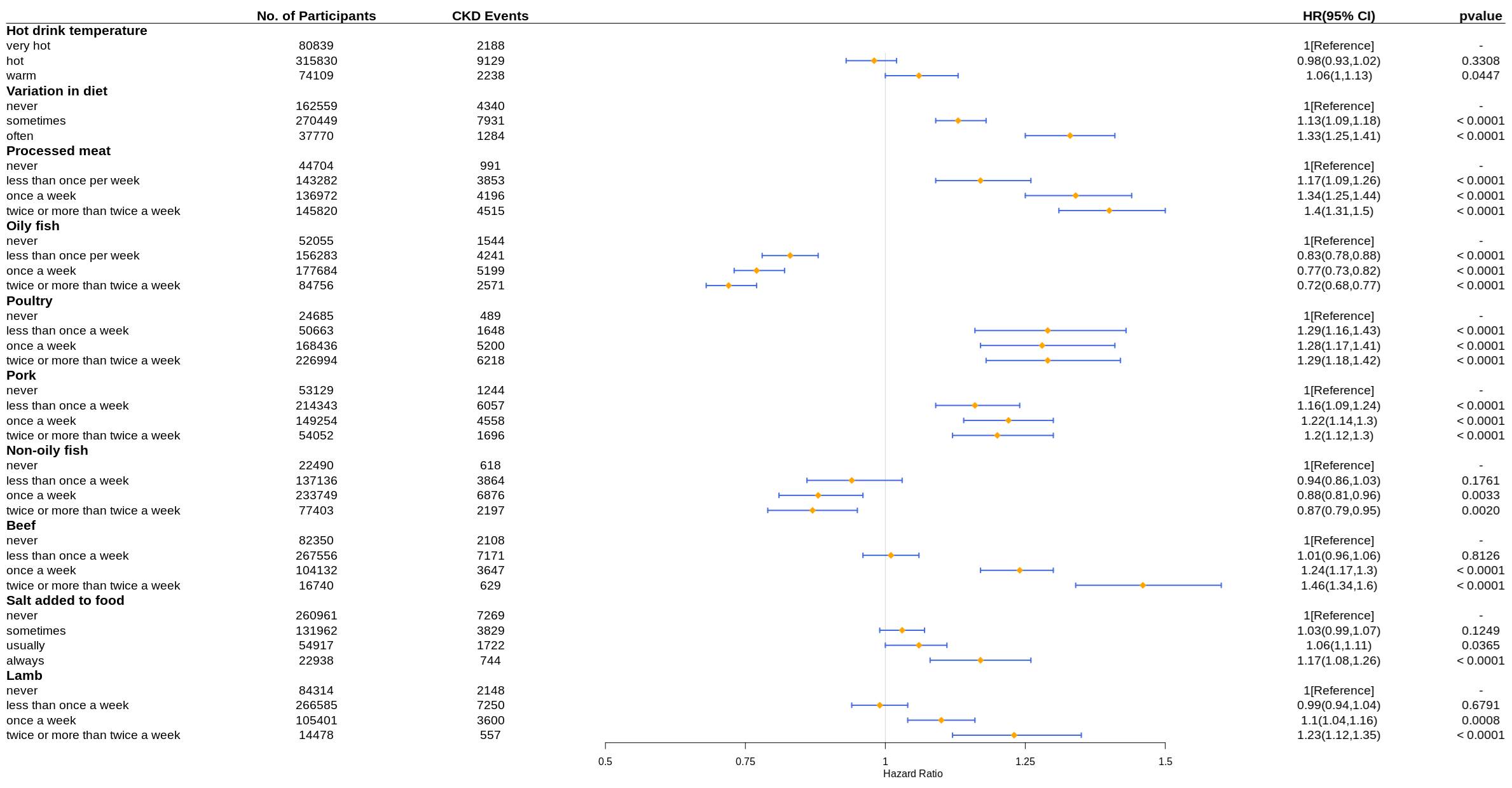


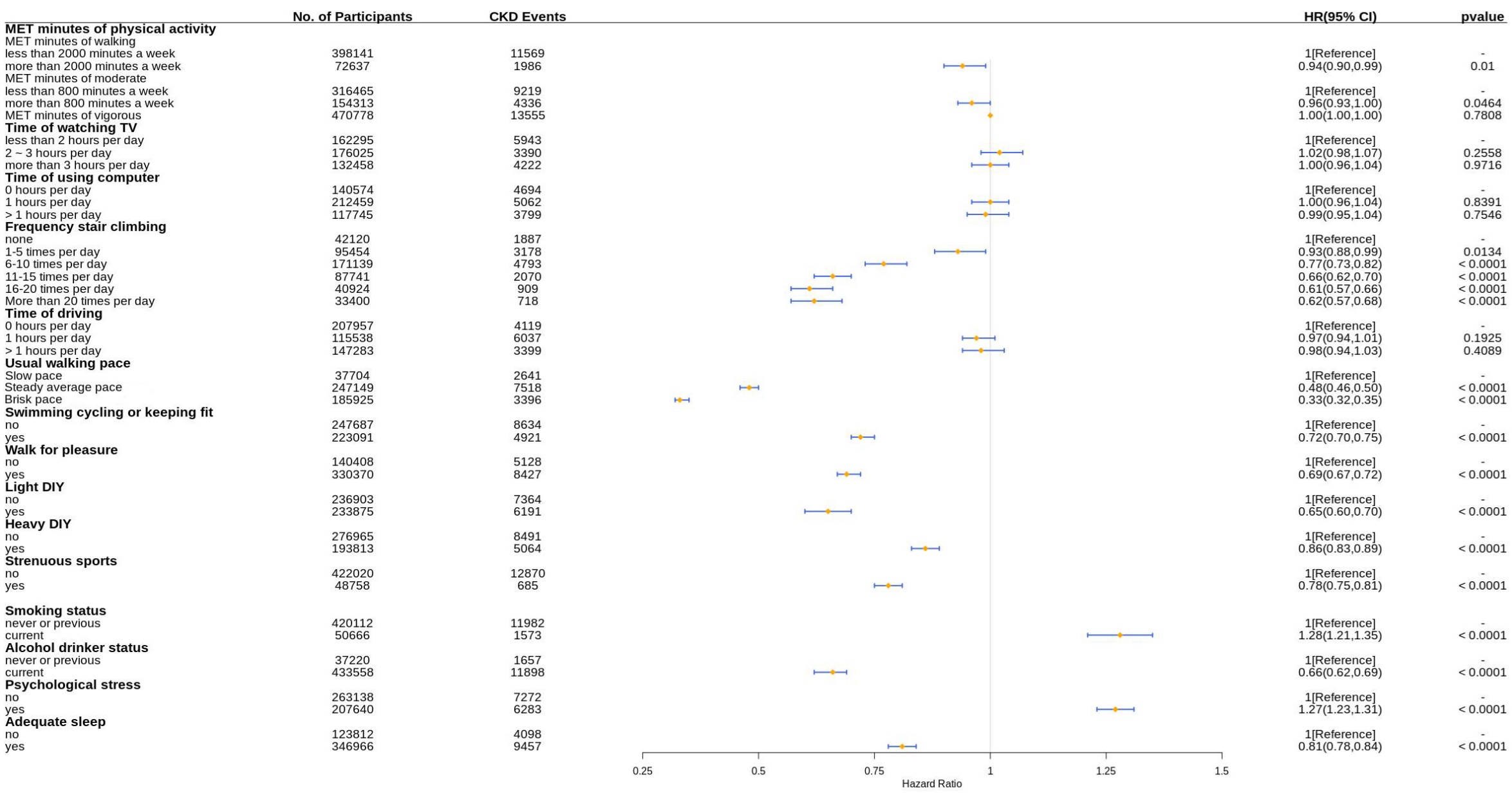


**Supplement figure 2. Hazard ratios for association between individual lifestyle factors and the incidence of CKD events.**

Moderate PA (physical activity) included walking upstairs, going the gym, jogging, energetic dancing aerobics, most sports, using heavy power tools and other physically demanding DIY & gardening. Vigorous PA included running, cycling uphill, carrying heavy furniture upstairs, martial arts, competitive sports or intensive exercise. Light DIY included pruning, watering the lawn; other exercises included swimming, cycling, keeping fit and bowling; Heavy DIY included weeding, lawn mowing, carpentry and digging.

Hazard ratios (HR) and 95% confidence intervals (CIs) were estimated with the use of a Cox proportional-hazards model. Multivariate analyses were adjusted for age and gender. The HR(95% CIs) of MET minutes for physical activity (walking, moderate, vigorous) were computed by continuous variable (1-unit increment), and the rest of variables were expressed as dichotomous or polytomous.
